# P03-14 Barriers to sports participation among adolescent girls from deprived neighbourhoods

**DOI:** 10.1093/eurpub/ckac095.050

**Published:** 2022-08-29

**Authors:** Cecilie Karen Ljungmann, Julie Hellesøe Christensen, Charlotte Demant Klinker, Charlotte Skau Pawlowski, Helene Rald Johnsen

**Affiliations:** 1 Steno Diabetes Center Copenhagen, Herlev, Denmark; 2 University of Southern Denmark, Odense, Denmark

**Keywords:** Adolescent girls, sport, deprived neighbourhoods, focus groups

## Abstract

**Background:**

A gender-based disparity in physical activity, where girls are less physically active than boys, is a persistent finding in the literature. Participation in organised sport plays a significant role for adolescents' physical activity habitsand provides an important means to achieve the recommended amount of daily physical activity. Nevertheless, participation in sport decreases with age, and to a higher degree among girls. Moreover, girls living in low socio-economic status neighbourhoods are even less represented in organised sport. This upholds a social inequality as a range of physical, mental and social benefits are associated with regular participation in sport. Empowering girls as experts on their own needs and preferences toward sport is critical. The aim of this qualitative study was to examine the experiences and perceived barriers to sports participation among adolescent girls living in low socio-economic status neighbourhoods.

**Methods:**

During July-October 2021 eleven semi-structured focus groups were carried out with adolescent girls (10-16 years) who were not engaged in organised sport. The participating girls (n = 44) were recruited through purposive sampling via four Danish schools placed in areas with high deprivation. A thematic analysis was conducted from verbatim transcripts using NVivo.

**Results:**

Six themes were identified; 1) Competing priorities, 2) Social aspects of sports participation, 3) Perceived lack of sporting abilities, 4) Feeling discomfort, 5) Gender stereotypes and 6) Physical Education as introduction to sport. Even though there were many similarities among the girls' perceived barriers to sports participation, the data showed variations in the way these barriers were experienced.

**Conclusions:**

The results provide insight into barriers to sports participation among adolescent girls living in low socio-economic status neighbourhoods. The findings correspond with other studies among adolescent girls in general. However, the findings among this underrepresented group will be useful to shed light on how interventions should be designed to promote sports participation among adolescent girls living in low socio-economic status neighbourhoods. As this is perceived as a hard-to-reach group, the findings will be a contribution to the field and are highly needed to illuminate the different perspectives on gender-based disparities that exist in relation to sports participation.

